# Unplanned hospitalizations in patients with locoregionally advanced head and neck cancer treated with (chemo)radiotherapy with and without prophylactic percutaneous endoscopic gastrostomy

**DOI:** 10.1186/s13014-020-01727-9

**Published:** 2020-12-14

**Authors:** Beat Bojaxhiu, Binaya K. Shrestha, Pascal Luterbacher, Olgun Elicin, Mohamed Shelan, Andrew J. S. Macpherson, Benjamin Heimgartner, Roland Giger, Daniel M. Aebersold, Kathrin Zaugg

**Affiliations:** 1 Department of Radiation Oncology, Inselspital, Bern University Hospital, University of Bern, Bern, Switzerland; 2 Department of Nuclear Medicine, Inselspital, Bern University Hospital, University of Bern, Bern, Switzerland; 3 Department of Visceral Surgery and Medicine, Division of Gastroenterology, Inselspital, Bern University Hospital, University of Bern, Bern, Switzerland; 4 Department of Otorhinolaryngology, Head and Neck Surgery, Inselspital, Bern University Hospital, University of Bern, Bern, Switzerland; 5 Department of Radiation Oncology, Stadtspital Triemli, Birmensdorferstrasse 497, 8063 Zurich, Switzerland

**Keywords:** Head and neck cancer, Percutaneous endoscopic gastrostomy, Morbidity, PEG, Radiotherapy

## Abstract

**Background:**

Current studies about percutaneous endoscopic gastrostomy (PEG) tube placement report equivalent patient outcomes with prophylactic PEG tubes (pPEGs) versus common nutritional support. Unreported was if omitting a pPEG is associated with an increased risk of complications leading to a treatment-related unplanned hospitalization (TRUH).

**Methods:**

TRUHs were retrospectively analyzed in patients with advanced head and neck squamous cell carcinoma (n = 310) undergoing (chemo)radiotherapy with (pPEG) or without PEG (nPEG).

**Results:**

In 88 patients (28%), TRUH was reported. One of the leading causes of TRUH in nPEG patients was inadequate oral intake (n = 16, 13%), and in pPEG patients, complications after PEG tube insertion (n = 12, 10%). Risk factors for TRUH were poor performance status, tobacco use, and surgical procedures.

**Conclusions:**

Omitting pPEG tube placement without increasing the risk of an unplanned hospitalization due to dysphagia, dehydration or malnutrition, is an option in patients being carefully monitored. Patients aged > 60 years with hypopharyngeal carcinoma, tobacco consumption, and poor performance status appear at risk of PEG tube-related complications leading to an unplanned hospitalization.

## Background

Curative intended radiotherapy (RT) with or without concomitant chemotherapy of patients with locoregionally advanced (Union for International Cancer Control [UICC] 7th edition: stage III–IVB) head and neck squamous cell carcinoma (HNSCC) may lead to malnutrition [1, 2], among other significant toxicities. An already existing dysphagia or odynophagia caused by the tumor can be aggravated by therapy-related inflammation, mucositis, and edema along the mucosal linings of the upper aero-digestive tract, as well as in the muscles of mastication and swallowing [3–5]. If this leads to grade 3 dysphagia according to Common Terminology Criteria for Adverse Events (CTCAE version 5.0) [6], feeding tube or total parenteral nutrition and/or hospitalization is indicated. Several feeding tube strategies can be used for this nutritional support (e.g. nasogastric tube, percutaneous endoscopic or percutaneous radiologic gastrostomy). Retrospective analyses examining the indication for PEG tube placement showed differing results, again making a conclusive statement challenging [7]. This is further complicated by the fact that a prophylactically inserted PEG (pPEG) is sometimes found subsequently not to be needed [8]. A comprehensive review found weak evidence concerning the pros and cons associated with pPEG placement and made a call for more prospective studies [9, 10]. One of the first prospective studies to include enough patients published its first results in 2012 [10], with an extended follow-up in 2017 [11]. The study compared the use of pPEGs with common nutritional support and enteral tube feeding (when considered necessary) inserted after the start of treatment reactively (rPEG). It resulted in no difference in swallowing function, tube dependence, and the prevalence of clinically relevant esophageal strictures. There was no difference in weight, body mass index (BMI), or overall survival (OS) between the groups. There was neither an advantage nor a disadvantage for a pPEG versus nPEG or a rPEG. However, in this study no complications leading to a hospitalization were reported in the nPEG group. Our study aimed to analyze if omitting a PEG tube in LAHNSCC patients was associated with an increased risk of complications leading to an unplanned hospitalization (UH), compared to patients receiving a PEG tube prophylactically.

## Methods

### Patients

In this retrospective single-center chart review, we identified a database of 310 consecutive UICC stage III–IVB HNSCC patients (except for nasopharyngeal and sinonasal sites) treated between 2007 and 2012 with primary or adjuvant chemo-RT with a curative intent. Ethics committee approval (Ref.-Nr. KEK-BE: 289/2014) was obtained for this study and it has been conducted in full accordance with ethical principles, including the World Medical Association Declaration of Helsinki (version 2002) and the additional requirements.

### Treatment and follow-up

Treatment strategies were based on institutional policies following the case-based multidisciplinary tumor board decision, as previously published [12, 13]. Patients who were first diagnosed before 2010 (UICC 6th edition) were re-staged according to the 7th edition during data acquisition. During this period, there was no algorithm as to which patient should be recommended for a pPEG. Prophylactic PEG placement was recommended to all patients based on the subjective evaluation of their general condition, expected radiation volume and side effects by the attending radiation oncologists. The cases in which a patient rejected a pPEG and the reasons of rejection were not systematically assessed. The planning and delivery of RT as well as the definitions of clinical target volume and planned target volume followed international recommendations [14–18]. The RT was administered with 2 Gy daily fractions using a volumetric modulated arc technique up to a total dose of 72 Gy for macroscopically detectable tumor, 66 Gy for postoperative positive or narrow resection margin(s), and the lymph node region(s) with pathological extracapsular extension. Elective nodal regions received 54 Gy. Sequential boosts were performed.

### Percutaneous endoscopic gastrostomy placement

Percutaneous endoscopic gastrostomy placements (pPEG and rPEG) were performed according to the pull method described by Ponsky et al [19]. Antibiotic prophylaxis with Amoxicillin/Clavulanate 1.2 g intravenously and a Freka PEG gastral 15 Ch/Fr EnFit (Fresenius Kabi, Switzerland) were routinely used.

### Definition of unplanned hospitalizations and follow-up

All complications leading to a hospitalization from the initial histopathological diagnosis to the last follow-up were recorded. Emergency or unplanned admissions were defined as unplanned hospitalizations (UHs). However, elective hospitalizations, including those due to socially or logistically difficult circumstances (e.g. long journey, initially poor general condition, etc.), were not analyzed. If an elective hospitalization was associated with a complication and therefore an extension to the planned length of stay, the time from that complication to discharge was defined as an UH. UHs were classified as being related to comorbidities, index HNSCC and recurrences, or cancer treatment. When a UH was related to cancer treatment, it was defined as a treatment-related UH (TRUH). In order not to have more than three endpoints and to enable a sound and simple statistical methodology, we analyzed only the first two UHs and thus only the first two consecutive TRUHs in patients who had multiple UHs. In the case of externally UH, additional information was obtained from the hospital where the emergency took place.

### Toxicities and the course of body weight

Toxicities and the course of body weight from the initial histopathological diagnosis to the last follow-up were recorded and graded according to CTCAE (version 5.0) [6]. The patient’s weight was recorded before, during, and after therapy. Changes were graded by CTCAE: grade 1, 5%–< 10% from baseline, intervention not indicated; grade 2, 10–≤ 20% from baseline, nutritional support indicated; grade 3 ≥ 20% from baseline, tube feeding, or total parenteral nutrition indicated. Symptoms of pain, dermatitis, mucositis, dysphagia, xerostomia, and osteoradionecrosis were assessed. Acute and late toxicities were defined as post-treatment-related complications occurring during and/or within 3 months or ≥ 3 months after commencing chemo-RT, respectively. Baseline pre-treatment tumor-related morbidity using the same criteria were also assessed.

### Statistical analysis

Patients were grouped as pPEG and nPEG. Patients who received rPEG were included in the nPEG group according to the intention-to-treat principle. The endpoints were defined as: first TRUH (TRUH1), second consecutive TRUH (TRUH2), and overall survival (OS). The start date of the first and second TRUH, and the date of death, were counted as the corresponding time points, respectively. Cox’s proportional hazard model was used to evaluate time-to-event endpoints, calculated from the date of histopathological diagnosis of the initial HNSCC. For multivariate analyses, backwards stepwise elimination was performed by including variables yielding *p* values ≤ 0.05 in univariate analyses. Actuarial time to event rates were depicted by Kaplan–Meier methodology. The chi-squared test was used to compare categorical variables. All tests were two-tailed. No adjustment was done for multiple testing. Due to the lack of concrete evidence or consensus regarding pre-treatment risk factors for feeding tube requirement to calculate and assign propensity scores, no matched-pair analyses were performed. Statistical analyses were performed with JMP (version 14.2.0; SAS Institute, Cary, NC, USA).

## Results

**Fig. 1:**
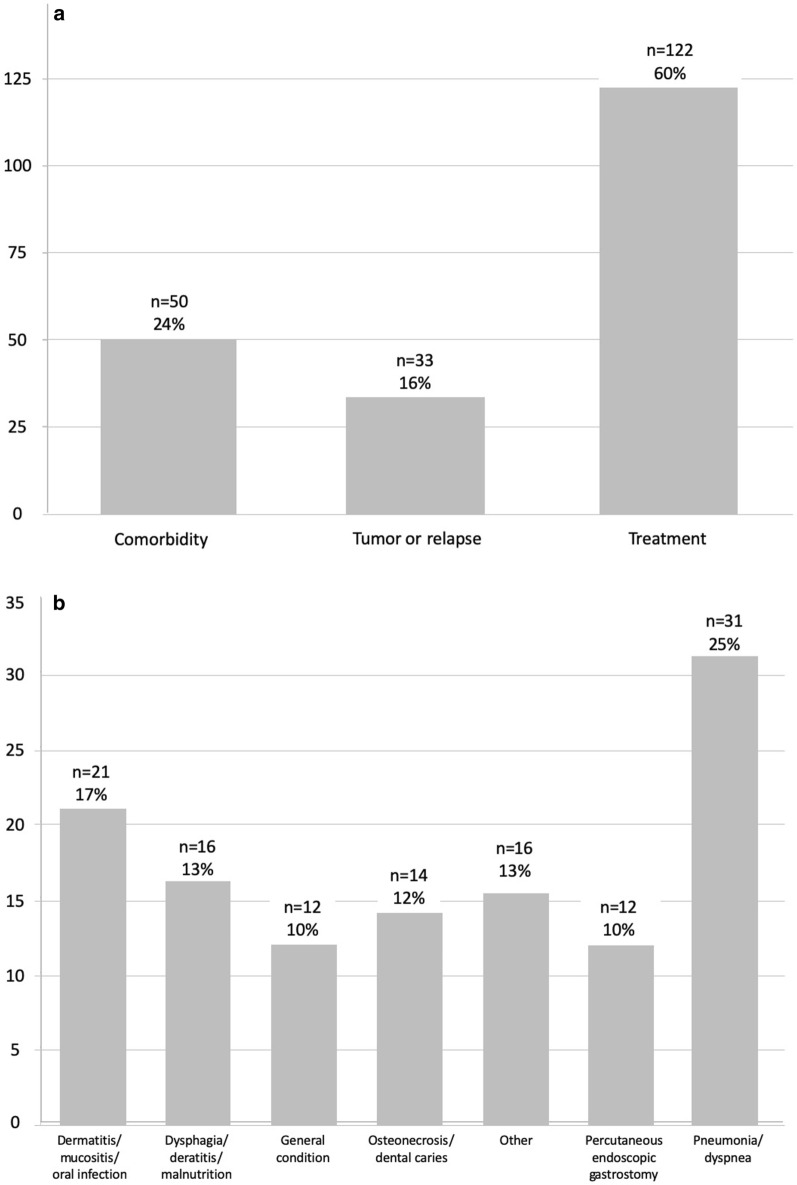
Overview of **a** causes leading to unplanned hospitalizations (UHs) and **b** to treatment related unplanned hospitalizations (TRUHs)

The median follow-up for the whole patient cohort was 32 months (range, 3–99 months). The median follow-up for the nPEG and pPEG group was 35 (range, 3–94 months), and 32 months (range, 3–99 months), respectively. Table 1 shows patient and disease characteristics. Compared with the nPEG group, the pPEG group had more patients aged 70–80 years, with a poor performance status (Eastern Cooperative Oncology Group performance status [ECOG PS] 2/3), hypopharyngeal tumors, and more advanced T and N stages. The patients with pPEG received concomitant chemotherapy more frequently and had less frequent grade 2 weight loss during therapy than nPEG patients. One hundred forty-one of the 310 patients (46%) had at least one UH: 88 (28%) were TRUH1, 34 (11%) comorbidity-related, and 19 (6%) relapse-related. Sixty-four patients (21%) had two consecutive UHs: 34 (11%) were TRUH2, 16 (5%) comorbidity-related, and 14 (5%) relapse-related. Table 2 and Fig. 1a show an overview of UHs; Table 3 and Fig. 1b show an overview of TRUHs.

**Table 1:** Patient and disease characteristics

Characteristic	All patients (n = 310)	pPEG (n = 175)	nPEG or rPEG (n = 135)	*P* value
Age at first diagnosis, years				
Median (range)	61 (20–94)	62 (20–83)	61 (40–94)	ns
≤ 60, n (%)	139 (44)	75 (43)	64 (47)	ns
> 60– ≤ 70, n (%)	111 (36)	36 (37)	47 (35)	ns
> 70– ≤ 80, n (%)	45 (15)	32 (18)	13 (10)	.035
> 80, n (%)	15 (5)	4 (2)	11 (8)	.029
Sex, n (%)				
Female	75 (24)	129 (74)	106 (79)	ns
Male	235 (76)	46 (26)	29 (21)	ns
ECOG performance status, n (%)				
0	112 (36)	56 (32)	56 (43)	ns
1	153 (50)	87 (50)	66 (50)	ns
2/3	40 (14)	30 (18)	9 (7)	.006
Missing, n	5	1	4	na
Median (range) baseline BMI, kg/m^2^	24.9 (16.8–38.7)	24.9 (16.8–38.6)	24.9 (17.6–36.8)	ns
Body weight loss during RT, CTCAE grade, n (%)				
0	135 (47)	92 (55)	43 (36)	.002
1	87(30)	48 (29)	39 (33)	ns
2	62 (22)	26 (15)	36 (30)	.004
3	2 (1)	1 (1)	1 (1)	ns
Missing, n	24	8	16	na
Smoking habits				
Never smoker	34 (13)	21 (13)	13 (12)	ns
Ex-smoker	75 (29)	47 (30)	28 (26)	ns
Current smoker	153 (58)	87 (56)	66 (62)	ns
Missing, n	48	20	28	na
Tobacco use, pack-years				
Median (range)	40 (0–150)	40 (0–150)	40 (0–120)	ns
> 40 (i.e. above median), n (%)	100 (44)	65 (46)	35 (41)	ns
Missing, n	83	34	49	ns
Alcohol abuse, n (%)				
No	85 (33)	54 (35)	31 (31)	ns
In the past	23 (9)	15 (10)	8 (8)	ns
Yes	147 (58)	85 (55)	62 (61)	ns
Missing, n	55	21	34	na
Tumor localization, n (%)				
Oral cavity	63 (20)	36 (21)	27 (20)	ns
Oropharynx	149 (48)	77 (44)	72 (53)	ns
Hypopharynx	44 (14)	33 (19)	11 (8)	.008
Larynx	39 (13)	16 (9)	23 (17)	ns
Multi-compartemental	15 (5)	13 (7)	2 (2)	.016
Tumor category, n (%)				
T1	25 (8)	9 (5)	16 (12)	.036
T2	95 (31)	42 (24)	53 (39)	.004
T3	102 (33)	59 (34)	43 (32)	ns
T4	88 (28)	65 (37)	23 (17)	< .001
Nodal category, n (%)				
N0	36 (12)	17 (10)	19 (14)	ns
N1	55 (18)	21 (12)	34 (25)	.004
N2	206 (66)	128 (73)	77 (57)	.003
N3	11 (4)	9 (5)	5 (4)	ns
UICC stage (7th edition), n (%)				
III	65 (21)	22 (13)	43 (32)	< .001
IVA	228 (74)	142 (81)	86 (64)	< .001
IVB	17 (5)	11 (6)	6 (4)	ns
Surgical interventions, n (%)				
Primary oncologic resection	78 (25)	42 (27)	36 (27)	ns
Neck dissection	214 (69)	121 (69)	93 (69)	ns
PEG tube placement, n (%)				
Prophylactic	175 (56)	175 (100)	0 (0)	na
Reactive	34 (11)	0 (0)	34 (25)	na
None	101 (33)	0 (0)	101 (75)	na
Median (range) duration of PEG dependency, days	266 (4–2969)	274 (40–2969)	231 (4–2554)	< .001
Chemotherapy, n (%)				
Concomitant	266 (86)	161 (92)	105 (78)	< .001
Neoadjuvant	33 (11)	21 (12)	12 (9)	ns

*BMI* body mass index, *CTCAE* Common Terminology Criteria for Adverse Events, *na* not applicable, *ns* not significant, *nPEG* no PEG, *PEG* percutaneous endoscopic gastrostomy, *pPEG* prophylactic PEG, *rPEG* reactive PEG, *RT* radiotherapy, *UICC* Union for International Cancer Control

**Table 2:** Overview of unplanned hospitalizations (n = 310)

UH	No. of patients (%)		All UHs, no. of events (%)
	UH1	UH2	
Any UH	141 (45)	64 (21)	205 events in 169 patients
Reason for UH			
Comorbidity-related	34 (11)	16 (5)	50 (24)
Alcoholism	4 (12)	0 (0)	4 (8)
Cardiopulmonary	9 (27)	3 (19)	12 (24)
Gastrointestinal	4 (12)	0 (0)	4 (8)
Infection	12 (35)	5 (31)	17 (34)
Other	5 (14)	8 (50)	13 (26)
Related to tumor or relapse	19 (6)	14 (4)	33 (16)
Treatment-related (TRUH)	88 (28)	34 (11)	122 (60)
Due to PEG	11 (13)	1 (2)	12 (10)
Due to surgery	3 (3)	1 (3)	4 (3)
Due to neoadjuvant CX	4 (4)	2 (6)	6 (5)
Due to radio-CX	70 (80)	29 (85)	99 (81)

*CX* chemotherapy, *TRUH* treatment-related unplanned hospitalization, *UH* unplanned hospitalization, *UH1* first UH event, *UH* second UH event

**Table 3:** Details of TRUHs (n = 310)

Reason for TRUH	All patients (n = 310)		pPEG (n = 175)		nPEG or rPEG (n = 135)	
	No	(%)	No	(%)	No	(%)
TRUH1 (n = 88)						
Dermatitis/mucositis/oral infection	18	(20)	13	(23)	5	(16)
Dysphagia/dehydration/malnutrition	15	(17)	7	(12)	8	(26)
General condition	8	(9)	5	(9)	3	(10)
Osteonecrosis/dental caries	8	(9)	5	(9)	3	(10)
Other	11	(11)	7	(13)	4	(13)
Percutaneous endoscopic gastrostomy	11	(14)	9	(16)	2	(6)
Pneumonia/dyspnea	17	(19)	11	(19)	6	(19)
Total	88	(28)	57	(33)	31	(23)
TRUH2 (n = 34)						
Dermatitis/mucositis/oral infection	3	(9)	3	(13)	0	(0)
Dysphagia/dehydration/malnutrition	1	(3)	1	(4)	0	(0)
General condition	4	(12)	2	(8)	2	(20)
Osteonecrosis/dental caries	6	(18)	3	(13)	3	(30)
Percutaneous endoscopic gastrostomy	3	(9)	0	(0)	1	(10)
Pneumonia/dyspnea	14	(41)	11	(46)	3	(30)
Other	3	(9)	4	(17)	1	(10)
Total	34	(11)	24	(14)	10	(7)
All TRUHs (n = 122)						
Dermatitis/mucositis/oral infection	21	(17)	16	(20)	5	(12)
Dysphagia/dehydration/malnutrition	16	(13)	8	(10)	8	(20)
General condition	12	(10)	7	(9)	5	(12)
Osteonecrosis/dental caries	14	(11)	8	(10)	6	(15)
Percutaneous endoscopic gastrostomy	12	(12)	11	(14)	1	(10)
Pneumonia/dyspnea	31	(25)	22	(27)	9	(22)
Other	13	(11)	9	(11)	4	(10)
Total number	122	(–)	81	(–)	41	(–)

*na* not applicable, *ns* not significant, *TRUH* treatment-related unplanned hospitalization, *TRUH1* first TRUH event, *TRUH2* second TRUH event, *nPEG* no percutaneous endoscopic gastrostomy, *pPEG* prophylactic percutaneous endoscopic gastrostomy, *rPEG* reactive percutaneous endoscopic gastrostomy

**Fig. 2:**
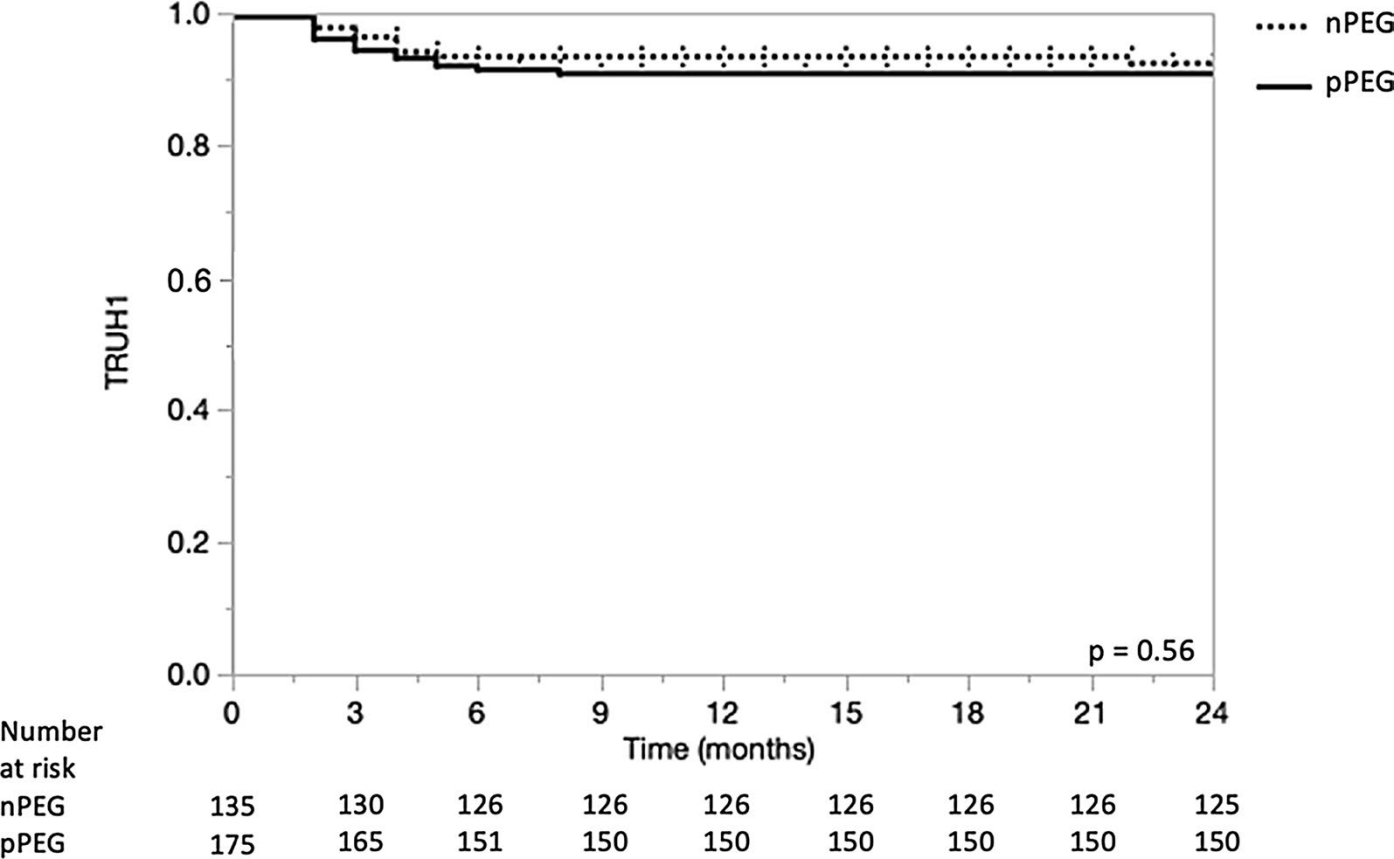
Kaplan–Meier curve comparing treatment-related unplanned hospitalizations (TRUHs) caused by prophylactic PEG tube installment (pPEG) versus TRUHs caused by omitting a PEG tube (dysphagia/dehydration/malnutrition)

Beside chemo-RT-related side effects (dermatitis, mucositis, infection, pneumonia, and dyspnea), the leading causes of TRUH in the nPEG group was dysphagia/dehydration/malnutrition. In the pPEG group, PEG complications were one of the leading causes for TRUH, besides chemo-RT-related side effects (Table 3). The comparison of TRUH1 regardless of its cause was 80% versus 70% (*p* = 0.09). The comparison of TRUH1 related to PEG complications or dysphagia/malnutrition/dehydration related events between nPEG and pPEG groups is shown in Fig. 2. There was no significant difference (*p* = 0.56). The same analysis was not repeated for TRUH2 due to the small number of events (n = 2). According to univariate analysis, risk factors for a TRUH were: poor ECOG PS (2/3), tobacco use > 40 pack-years (i.e. above the median), and surgical procedures ([bilateral] neck dissection, tracheostomy, and pPEG). In multivariate analysis, tobacco use > 40 pack-years, bilateral neck dissection, and poor ECOG PS (2/3) remained as independent risk factors for TRUH. (Table 4). We investigated possible risk factors for a PEG-associated event in a subgroup analysis, which revealed that tumor localization to hypopharynx (*P* = 0.0183), active tobacco consumption (*P* = 0.0009), tobacco use > 40 pack-years (*P* = 0.0001), poor ECOG PS (2/3) (*P* = 0.0418), and age > 60 years (*P* = 0.0352) were risk factors in the univariate analysis (data not shown). Overall survival at 3 years for the entire, nPEG, and pPEG group was 70%, 67%, and 73%, respectively. Overall survival was associated with age, ECOG PS 2–3, tumor localization to the oropharynx and hypopharynx, neck dissection, rPEG, and baseline BMI (Table 4 and Additional File 1).

**Table 4:** Univariate and multivariate analysis (Cox proportional-hazard)

Variable	TRUH1		TRUH2		OS	
	HR (95% CI)	*P* value	HR (95% CI)	*P* value	HR (95% CI)	*P* value
Univariate						
Age, years						
≤ 60	0.76 (0.48–1.18)	.21	0.74 (0.35–1.56)	.42	0.63 (0.42–0.96)	**.0288**
> 60–≤ 70	1.00 (0.64–1.58)	.99	1.07 (0.51–2.67)	.86	0.92 (0.60–1.40)	.6884
> 70–≤ 80	1.43 (0.82–2.50)	.20	1.59 (0.65–3.91)	.30	1.40 (0.84–2.34)	.1937
> 80	1.52 (0.61–3.75)	.35	0.80 (0.11–5.85)	.82	4.25 (2.26–7.99)	**.0001**
ECOG PS at first consultation, (vs other PS)						
0	0.95 (0.60–1.51)	.83	0.89 (0.41–1.91)	.76	0.64 (0.41–1.00)	**.0475**
1 (vs others)	0.73 (0.47–1.14)	.16	0.59 (0.28–1.26)	.17	1.05 (0.71–1.58)	.7939
2/3 (vs others)	**1.99 (1.15–3.45)**	**.01**	**2.77 (1.23–6.27)**	**.01**	2.01 (1.21–3.37)	**.0061**
Alcohol abuse						
Active	1.16 (0.71–1.88)	.55	0.81 (0.36–1.83)	.60	1.23 (0.78–1.94)	.3783
Active or in past	1.59 (0.92–2.75)	.09	1.17 (0.48–2.85)	.72	1.33 (0.82–2.16)	.2475
> 2 units a day (= median)	1.44 (0.81–2.57)	.21	0.84 (0.30–2.35)	.73	1.06 (0.63–1.79)	.8203
Smoking habits						
Current smoker	1.13 (0.70–1.82)	.61	0.94 (0.41–2.13)	.87	1.06 (0.68–1.66)	.7832
Current or ex-smoker	1.68 (0.74–3.92)	.20	3.39 (0.46–25.1)	.20	1.14 (0.59–2.21)	.7022
> 40 pack-years (= median)	**1.94 (1.17–3.20)**	**.01**	1.98 (0.81–4.85)	.12	1.01 (0.63–1.63)	.9615
Tumor localization, yes (vs no)						
Oral cavity	1.06 (0.62–1.81)	.83	0.81 (0.31–2.12)	.67	0.80 (0.48–1.36)	.409
Oropharynx	0.75 (0.48–1.17)	.19	0.75 (0.36–1.56)	.43	0.63 (0.42–0.95)	**.026**
Hypopharynx	1.43 (0.81–2.50)	.20	2.00 (0.86–4.69)	.10	1.81 (1.11–2.93)	**.0149**
Larynx	1.21 (0.66–2.23)	.53	0.79 (0.24–2.60)	.69	1.39 (0.81–2.38)	.2251
Mixed	0.80 (0.25–2.52)	.69	1.60 (0.38–6.73)	.56	1.81 (0.74–4.48)	.1878
Surgery, yes (vs no)						
Primary oncologic surgery	0.72 (0.42–1.24)	.22	0.47 (0.16–1.34)	.15	0.79 (0.49–1.29)	.3395
Neck dissection	0.69 (0.44–1.07)	.09	**0.47 (0.23–0.96)**	**.03**	0.66 (0.44–0.99)	**.0438**
Bilateral ND	**1.69 (1.08–2.64)**	**.00**	1.75 (0.84–3.67)	.13	0.92 (0.58–1.45)	.7161
Tracheostomy	**1.78 (1.15–2.77)**	**.01**	**2.58 (1.24–5.35)**	**.01**	1.49 (0.98–2.25)	.0594
pPEG	1.46 (0.93–2.30)	.09	**2.56 (1.09–5.99)**	**.02**	1.24 (0.82–1.86)	.3003
rPEG	2.54 (1.48–4.33)	**.00**	1.75 (0.67–4.58)	.25	2.07 (1.25–3.43)	**.0038**
Chemotherapy, yes (vs no)						
Neoadjuvant	1.53 (0.83–2.83)	.57	**2.82 (1.20–6.60)**	**.01**	1.26 (0.69–2.31)	.4535
Concomitant	1.22 (0.61–2.43)	.16	2.08 (0.50–8.76)	.30	0.59 (0.35–1.02)	.054
Baseline BMI, kg/m^2^						
≤ 18.5	1.11 (0.27–4.54)	.89	1.46 (0.20–10.8)	.71	3.42 (1.23–9.50)	**.0115**
> 18.5–< 25	0.70 (0.41–1.22)	.20	0.73 (0.30–1.73)	.46	0.94 (0.56–1.58)	.8135
≥ 25	1.39 (0.81–2.38)	.22	1.29 (0.55–3.02)	.55	0.88 (0.53–1.47)	.6231
Multivariate						
Model 1 for TRUH1						
ECOG 2/3	1.73 (0.93–3.23)	.10	–	–	–	–
> 40 pack-years	1.94 (1.17–3.20)	**.01**	–	–	–	–
Bilateral ND	1.69 (1.02–2.78)	**.04**	–	–	–	–
Model 2 for TRUH2						
ECOG 2/3	–	–	2.77 (1.23–6.27)	**.03**	–	–
pPEG	–	–	2.06 (0.87–4.91)	.09	–	–
Neoadjuvant CX	–	–	3.27 (1.38–7.74)	**.02**	–	–
Model 3 for OS						
Age > 80 years	–	–	–	–	4.25 (2.26–7.99)	**.00**
ECOG 2/3	–	–	–	–	1.64 (0.94–2.85)	.10
rPEG	–	–	–	–	1.95 (1.18–3.25)	**.02**

*P* values in bold are significant at *P* < 0.05

*BMI* body mass index, *CI* confidence interval, *ECOG PS* Eastern Cooperative Oncology Group performance status, *HR* hazard ratio, *OS* overall survival, *TRUH* treatment-related unplanned hospitalization, *TRUH1* first TRUH event, *TRUH2* second TRUH event, *pPEG* prophylactic percutaneous endoscopic gastrostomy, *rPEG* Reactive percutaneous endoscopic gastrostomy, *ND* neck dissection, *CX* chemotherapy

## Discussion

In a relatively large cohort of 310 patients with locoregionally advanced HNSCC compared with previously published studies [7, 9, 20–23], we retrospectively analyzed whether omitting a PEG compared to prophylactic PEG insertion is associated with an increased risk of complications leading to a TRUH. Although the institutional policy was to offer pPEG to all patients with locoregionally advanced HNSCC, physicians were less keen on insisting that patients with a possibly lower risk profile should receive a pPEG. Moreover, some patients refused the pPEG regardless of their risk profile. Therefore, pPEG placement tended to be used more frequently in patients with a higher risk profile and worse prognosis (comprising general condition, tumor size, age, hypopharyngeal tumor localization; Table 1). In the nPEG group, apart from (chemo)-RT-related side effects, dysphagia/dehydration/malnutrition (n = 8; 20%) was the most frequent cause of TRUH, whereas in the pPEG group, apart from (chemo)-RT-related side effects, PEG-related complications frequently led to TRUH (n = 11; 14%). There was no difference in TRUH caused by PEG complications or dysphagia/malnutrition/dehydration after pPEG versus nPEG (*p* = 0.56). PEG tube placement is associated with the risk of complications; however, there is a great deal of variability in the reported incidence of such complications [11, 20, 24–29]. The difference in the incidences of complications is partly due to the various definitions and populations analyzed. For example, complications are more likely to occur in older patients with comorbidities, especially those with an infection or history of aspiration [30]. Compared with the publication of Silander et al. [10], our rate of PEG-related complications is relatively high (14% vs 1%); however, it is relatively low compared with a prospective study reporting complication rates at 2 weeks and 2 months (39% and 27%, respectively) [24]. We hypothesize that our pPEG cohort is a different, more fragile patient population that tends to have more complications compared with that studied by Silander et al. [10] and our nPEG population. Furthermore, we suspect that patients—like those studied by our Swedish colleagues [10]—who are willing to be included and randomized in a study, are more compliant than the patients with HNSCC seen in our everyday practice, over two-thirds of whom have a positive history of alcohol abuse and more than 87% a positive history of tobacco use [31]. Patients with severe clinical and psychosocial impairment and fewer economic resources are more likely to experience treatment compliance problems [31]. There is an increasing incidence of oropharyngeal cancer, especially in younger patients, and a decrease in the previously known risk factors for HNSCC of smoking and alcohol use [32, 33]. Previously, typical patients with HNSCC tended to be heavy drinkers or smokers; however, human papillomavirus (HPV)-associated HNSCC in younger, fitter, and possibly more compliant patients increasingly represent the majority of at least oropharyngeal disease [34]. This interesting aspect should be kept in mind before considering that in our entire cohort, up to one-fifth of patients had a TRUH besides the (chemo)-RT-induced TRUH—due to dysphagia/dehydration/malnutrition (20%) in the nPEG group or postoperatively after PEG insertion (12%) in the pPEG group. The physician and patient have to face the additional risks associated with an invasive procedure, such as PEG tube placement, or those arising from not performing a supportive surgical procedure to allow sufficient oral intake, such as dysphagia/dehydration/malnutrition.

Further differences between our cohort and the Swedish study [10, 11] can be seen with regard to weight loss, BMI, and OS between the pPEG and nPEG groups. The increased weight loss and BMI differences during RT in the nPEG versus pPEG groups could be explained not only by the greater compliance of patients but also by the prospective setting—and therefore thorough monitoring by nutrition counselors in the nPEG cohort—of the Silander et al. trial [10]. Nutrition counselors were not systematically involved in the treatment of our patients, and some patients categorically refused nutrition counseling. The higher risk profile in the pPEG group more easily explains the OS difference versus the nPEG group (tumor size, age, tumor localization; Table 1), as OS is known to be worse in patients with larger primary tumors and hypopharyngeal tumor [35–38]. Other limitations of our study, apart from the different risk profiles of the nPEG and pPEG groups, include its retrospective nature, the lack of stratification according to HPV status, and the presence of some patients treated with surgery before RT.

With future changes in the HNSCC population, therapy regimens, and side-effect profile according to the HPV status, further analyses of the indication for a PEG is necessary [39].

## Conclusions

Our retrospective analysis shows that omitting a prophylactic PEG does not lead to more unplanned hospitalizations compared to patients receiving a PEG tube before start of chemoradiation. Patients with a hypopharyngeal carcinoma, active tobacco consumption, more than 40 pack-years of smoking history, and poor ECOG PS seem to be at risk of PEG tube-related UH. Prospective trials about pPEG, especially for oropharyngeal carcinoma and its future results concerning de-escalation, are warranted.

## Supplementary Information

**Additional file 1.** Complete multivariate analyses.

## Data Availability

The datasets used and/or analyzed during the current study are available from the corresponding author on reasonable request.

## Abbreviations

PEG: Percutaneous endoscopic gastrostomy
pPEG: Prophylactic percutaneous endoscopic gastrostomy
nPEG: No prophylactic percutaneous endoscopic gastrostomy
rPEG: Reactive percutaneous endoscopic gastrostomy
UH: Unplanned hospitalization
TRUH: Treatment-related unplanned hospitalization
RT: Radiotherapy
UICC: Union for International Cancer Control
HNSCC: Head and neck squamous cell carcinoma
CTCAE: Common Terminology Criteria for Adverse Events
OS: Overall survival
BMI: Body mass index
na: Not applicable
ns: Not significant
CI: Confidence interval
ECOG PS: Eastern Cooperative Oncology Group performance status
HR: Hazard ratio
ND: Neck dissection
CX: Chemotherapy
