# Supplementary material for: Unplanned hospitalizations in patients with locoregionally advanced head and neck cancer treated with (chemo)radiotherapy with and without prophylactic percutaneous endoscopic gastrostomy
Source: Radiat Oncol. 2020 Dec 14;15:281. doi: 10.1186/s13014-020-01727-9 (PMC7737384; doi:10.1186/s13014-020-01727-9)
Supplement: Supplementary file 1 — Additional file 1. Complete multivariate analyses. [file 13014_2020_1727_MOESM1_ESM.docx]

**SUPPLEMENTARY TABLE 1:** Complete multivariate analysis

| **Variable** | **TRUH1** | | **TRUH2** | | **OS** | |
| --- | --- | --- | --- | --- | --- | --- |
|  | **HR (95% CI)** | ***P* value** | **HR (95% CI)** | ***P* value** | **HR (95% CI)** | ***P* value** |
| *Multivariate analysis* | | | | | | |
| **Age, years** |  |  |  |  |  |  |
| ≤60 | n.a. | n.a. | n.a. | n.a. | 0.94 (0.53-1.67) | .83 |
| >60–≤70 | n.a. | n.a. | n.a. | n.a. | n.a. | n.a. |
| >70–≤80 | n.a. | n.a. | n.a. | n.a. | n.a. | n.a. |
| >80 | n.a. | n.a. | n.a. | n.a. | 0.87 (0.25-3.04) | .83 |
| **ECOG PS at first consultation**, **(vs other PS)** | | |  |  |  |  |
| 0 | n.a. | n.a. | n.a. | n.a. | 0.77 (0.41-1.45) | .42 |
| 1 (vs others) | n.a. | n.a. | n.a. | n.a. | n.a. | n.a. |
| 2/3 (vs others) | 1.64 (0.86-3.11) | .13 | 2.37 (1.02-5.60) | **0.05** | 3.24 (1.40-7.52) | **.01** |
| **Alcohol abuse** |  |  |  |  |  |  |
| Active | n.a. | n.a. | n.a. | n.a. | n.a. | n.a. |
| Active or in past | n.a. | n.a. | n.a. | n.a. | n.a. | n.a. |
| >2 units a day (=median) | n.a. | n.a. | n.a. | n.a. | n.a. | n.a. |

| **Smoking habits** |  |  |  |  |  |  |
| --- | --- | --- | --- | --- | --- | --- |
| Current smoker | n.a. | n.a. | n.a. | n.a. | n.a. | n.a. |
| Current or ex-smoker | n.a. | n.a. | n.a. | n.a. | n.a. | n.a. |
| >40 pack-years (=median) | 2.08 (1.25-3.46) | **.01** | n.a. | n.a. | n.a. | n.a. |
| **Tumor localization, yes (vs no)** | |  |  |  |  |  |
| Oral cavity | n.a. | n.a. | n.a. | n.a. | n.a. | n.a. |
| Oropharynx | n.a. | n.a. | n.a. | n.a. | 0.63 (0.34-1.17) | .14 |
| Hypopharynx | n.a. | n.a. | n.a. | n.a. | 2.90 (1.39-6.07) | **.01** |
| Larynx | n.a. | n.a. | n.a. | n.a. | n.a. | n.a. |
| Mixed | n.a. | n.a. | n.a. | n.a. | n.a. | n.a. |
| **Surgery, yes (vs no)** |  |  |  |  |  |  |
| Primary oncologic surgery | n.a. | n.a. | n.a. | n.a. | n.a. | n.a. |
| Neck dissection | n.a. | n.a. | 0.35 (0.14-0.89) | **.03** | 0.66 (0.37-1.17) | .16 |
| Bilateral ND | 1.08 (0.57-2.07) | .80 | n.a. | n.a. | n.a. | n.a. |
| Tracheostomy | 1.81 (0.94-3.50) | .08 | 3.10 (1.32-7.28) | **.01** | n.a. | n.a. |
| pPEG | n.a. | n.a. | 1.62 (0.66-3.95) | .29 | n.a. | n.a. |
| rPEG | 2.11 (1.11-4.00) | **.02** | n.a. | n.a. | 2.08 (0.97-4.47) | .06 |
| **Chemotherapy, yes (vs no)** | |  |  |  |  |  |
| Neoadjuvant | n.a. | n.a. | 1.89 (0.71-5.07) | .21 | n.a. | n.a. |
| Concomitant | n.a. | n.a. | n.a. | n.a. | n.a. | n.a. |
| **Baseline BMI, kg/m^2^** |  |  |  |  |  |  |
| ≤18.5 | n.a. | n.a. | n.a. | n.a. | 4.98 (1.96-14.7) | **.00** |
| >18.5–<25 | n.a. | n.a. | n.a. | n.a. | n.a. | n.a. |
| ≥25 | n.a. | n.a. | n.a. | n.a. | n.a. | n.a. |
|  |  |  |  |  |  |  |

Note: *P* values in bold are significant at *P*<0.05.

Abbreviations: BMI, body mass index; CI, confidence interval; ECOG PS, Eastern Cooperative Oncology Group performance status; HR, hazard ratio; OS, overall survival; TRUH, treatment-related unplanned hospitalization; TRUH1, first TRUH event; TRUH2, second TRUH event; pPEG, prophylactic percutaneous endoscopic gastrostomy; rPEG, Reactive percutaneous endoscopic gastrostomy; ND, neck dissection; CX, chemotherapy; n.a., not applicable
